# Investigation of the Effects of 2.45 GHz Near-Field EMF on Yeast

**DOI:** 10.3390/antiox14070820

**Published:** 2025-07-03

**Authors:** Boyana Angelova, Momchil Paunov, Meglena Kitanova, Gabriela Atanasova, Nikolay Atanasov

**Affiliations:** 1 Department of Biophysics and Radiobiology, Faculty of Biology, Sofia University “St. Kliment Ohridski”, 1164 Sofia, Bulgaria; m_paunov@uni-sofia.bg; 2 Department of Genetics, Faculty of Biology, Sofia University “St. Kliment Ohridski”, 1164 Sofia, Bulgaria; m.kitanova@uni-sofia.bg; 3 Department of Communication and Computer Engineering, South-West University “Neofit Rilski”, 2700 Blagoevgrad, Bulgaria; gatanasova@swu.bg (G.A.); natanasov@swu.bg (N.A.)

**Keywords:** *Saccharomyces cerevisiae*, electromagnetic pollution, RF-EMF, microwaves, heating, cell membrane permeability, antioxidant capacity, glutathione, comet assay, non-thermal effects

## Abstract

The study of the effects of 2.45 GHz electromagnetic fields on the health and safety of people and organisms as a whole is essential due to their widespread use in everyday life. It is known that they can cause thermal and non-thermal effects—at the molecular, cellular and organismal level. Yeast suspensions were treated with 2.45 GHz microwave radiation in the near-field of antenna at two distances (2 and 4 cm) and two time periods (20 and 60 min)—setups resembling the use of mobile devices. The release of UV-absorbing substances from the cells was studied as an indicator of membrane permeabilization, total intracellular antioxidant activity and reduced glutathione were determined, and a comet assay for damage to the DNA was performed. A correlation between reduced antioxidants and increased membrane permeability during EMF treatment was observed at a distance of 2 cm for 20 min, suggesting the presence of oxidative stress, while a similar effect was not observed with conventional heating. Slightly increased membrane permeability was observed after irradiation for 60 min at a distance of 4 cm, but this was not related to the antioxidant status of the cells. A trend towards increased DNA damage was observed under both conditions.

## 1. Introduction

The modern technological world creates more and more conveniences for people by offering them a wide range of various electronic devices that emit radio- or microwaves—non-ionizing electromagnetic fields (EMFs). Even though they carry low energy, their elevated levels in the environment are known as electromagnetic pollution and constitute a relatively new stress factor for the biosphere as a whole and a potential health risk for the population in particular. The physical properties that could determine the biological outcome of an EMF interaction are the frequency of the EMF wave, its polarization, the exposure duration, the geometry and structure of the object and the absorbed dose. A major parameter used to quantify the radio- and microwave frequency EMF energy intercepted by biological objects is the Specific Absorption Rate (SAR), W/kg, given as the electromagnetic wave power (in W) that has been absorbed by 1 kg of biological tissue. Another important parameter is the distance between the object and the emitting source, not only because it determines the wave intensity at the spot of the object but due to the fact that the physical properties of the EMF are quite different close (near-field) and farther from it (far-field—classic electromagnetic wave).

There are a number of scientific studies on the effects of microwave EMFs with different frequencies and intensities on microorganisms, plants, animals and humans [1]. The biological effects caused by microwave EMFs can be divided into thermal and non-thermal. Thermal effects are a result of the absorption of microwaves at the molecular level, which leads to particle vibrations and consequently heats the matter [2]. Evidence for non-thermal effects of electromagnetic radiation comes from studies with microorganisms, where a larger number of them was destroyed during irradiation than when heated conventionally (i.e., by a heater) to the same temperature [3]. There are also data showing the increased growth of cultures not related to heating [4]. The mechanisms behind these effects are not fully understood, but it is suggested that they are related to changes in the secondary and tertiary structure of functionally active proteins [5,6,7].

One of the most commonly used frequency bands for domestic, industrial, scientific and medical purposes is 2.45 GHz. The effect of 2.45 GHz microwave radiation on histology and lipid peroxidation levels in rats was studied [8]. The animals were exposed to radiation for 35 days, 2 h per day, with a SAR of 0.14 W/kg. Significant levels of lipid peroxidation were recorded in the liver, brain and spleen. Histological changes were found in the brain, liver, testes, kidneys and spleen compared to the control group. Karatopuk found that prolonged irradiation of rats (30 days for one hour per day) with a 2.45 GHz EMF led to endothelial damage [9]. Single- and double-stranded DNA breaks were detected by a comet assay in brain cells from rats irradiated for two hours with a 2.45 GHz EMF and a SAR ranging from 0.6 to 1.2 W/kg [10]. The effects were prevented by treatment with antioxidants, suggesting a free radical-related mechanism. The effects of 2.45 GHz electromagnetic radiation on the cell redox status were studied using human SH-SY5Y neuroblastoma cells, which had been differentiated into neuron-like cells and peripheral blood mononuclear cells [11]. They were treated for 2, 24 and 48 h. Cell viability was reduced after 24–48 h. Reactive oxygen species (ROS) levels were significantly increased in the exposed cells compared to the controls at each treatment period. The results showed that neuron-like cells were more prone to develop oxidative stress compared to PBMCs after exposure to a 2.45 GHz EMF and undergo activation of an early antioxidant defense response.

The effect of EMFs with a frequency band between 1 and 5.9 GHz on *Saccharomyces cerevisiae* yeast was monitored [12]. A decrease in cell viability was observed at all applied frequencies. The effect of the treatment directly depended on the distance between the antenna and the sample, that is, the intensity of the radiation. Transmission electron microscopy showed that the EMF led to a disruption of the integrity of the cell membrane. When exposing various microorganisms, including yeast, to an EMF with a frequency of 2.45 GHz and an amplitude of the electric field of 9.3 kV/m, no effect on cell survival was observed [13]. However, an increased permeability of the cell membrane for propidium iodide and dextran particles of different sizes was found. Propidium iodide entry was observed in microwave-treated *Mycobacterium smegmatis* cells, but not in cells conventionally heated to the same temperature as that reached by irradiation. Permeability to fluorescently labeled dextrans of 3 kDa was observed in all cell types, but larger dextran particles (70 kDa) were unable to enter the cells. DNA release from the cells was also observed. Abed et al. found that exposure of *S. cerevisiae* cells to microwave radiation at a frequency of 2.45 GHz and 90 W for 1 min resulted in cell deformation, delayed fermentation and failure to synthesize toxins [14]. Low-intensity microwave radiation (dose up to 1.6 W/g and duration up to 120 s) promoted growth and caused reversible permeabilization of the cell membrane without damaging the cells of *Brettanomyces custersii* yeast, isolated from spontaneously fermented rice paste [15]. Higher intensities led to yeast death, mainly due to irreversible permeabilization and the release of electrolytes and larger vital molecules from the cells.

Yeasts are widely used as a model organism, as they combine the experimental advantages of microorganisms and the characteristic features of higher eukaryotes. A comparative analysis of the yeast and human genomes shows conservatism in the genetic pathways responsible for the cellular response to various stress factors, including oxidative stress [16].

Although there are a number of studies on the effects of a 2.45 GHz EMF on various experimental subjects—from tissue cultures to whole organisms—the results obtained are not unambiguous. While some authors have reported serious damage at various levels of organization, others have not found any significant effects, especially at low SAR levels. The reason for the discrepancy in results may be a combination of various factors such as differences in irradiation parameters; the biological system and its degree of differentiation and cell cycle phase; the sensitivity of the methods used; and temperature control during treatment. There is a real need for further studies and more rigorously standardized experimental protocols to provide a clear assessment of biological risk. Data are particularly lacking in certain models such as yeast, which opens up opportunities for new research. There are no documented studies in the scientific literature where yeasts were irradiated with a 2.45 GHz EMF followed by a comet assay to determine DNA damage. There is also no information on the antioxidant status of cells after irradiation at this frequency.

The aim of our study was to investigate the short-term effects of near-field 2.45 GHz EMF exposure, similar to the use of mobile devices, on yeast, with a focus on cell membrane permeability, to expand existing knowledge in this area, as well as to gain new insights into antioxidant levels and DNA integrity.

## 2. Materials and Methods

### 2.1. Yeast Culture Conditions and Sample Preparation

The biological object of this study was yeast cells of the species *Saccharomyces cerevisiae* Meyen *ex* E.C. Hansen, strain U1, reference number 584 of NBIMCC (National Bank for Industrial Microorganisms and Cell Cultures, University of Chemical Technology and Metallurgy, Sofia, Bulgaria) [17]. The yeast cells were kindly provided as solid culture, whose medium contained 10 g of yeast extract, 20 g of peptone, 20 g of dextrose (YPD) and 15 g of agar diluted in distilled water (d. w.) to 1000 mL, in test tubes by the Department of General and Industrial Microbiology, Faculty of Biology, Sofia University “St. Kliment Ohridski”. A liquid culture was prepared by resuspending the agar culture with 1 mL of YPD broth, same as above but without agar, followed by inoculating 0.5 mL of this suspension in 50 mL of the YPD medium and incubating at 30 °C and 250 RPM in orbital shaker–incubator ES-20 Grant-bio (Grant Instruments Europe B.V., Amsterdam, The Netherlands) for 24 h. That culture was used as the first inoculum (1.5 mL) in a consecutive series of 24 h cultivations in 60 mL YPD broth (30 °C, 250 RPM) to keep the yeast cells vital and in a uniform physiological state throughout the experiments. All cultures were stored at 10 °C after cultivation. Before each experiment, fresh cultures were prepared and used directly (without storing) as an inoculum (1.5 mL) for the final cultivation (60 mL of YPD broth, 30 °C, 250 RPM, 24 h) to obtain the yeast cells for treatment. The *S. cerevisiae* cells from those cultures were washed with d. w. by centrifugation at 4200 RPM for 10 min (MPW-360, Mechanika Precyzyjna, Warsaw, Poland) and subsequent resuspending, three times in total, and concentrated finally to a 100 mg FW/mL suspension (in d. w.). The prepared suspension was distributed in 2 mL tubes which were used as samples for treatment.

### 2.2. Treatment Conditions

The 2 mL 100 mg FW/mL yeast suspensions were transported to a semi-anechoic chamber where they were irradiated with a 2.45 GHz continuous wave EMF with 3 exposure setups, combining a 2 or 4 cm distance to the antenna and a 20 or 60 min treatment duration, as outlined below:

1. 2 cm and 20 min;
2. 4 cm and 20 min;
3. 4 cm and 60 min.

The EMF was emitted by a dipole antenna with a length of 3 cm and an output power level of −10 dBm (100 μW) generated by a SMB-100A generator (Rohde & Schwarz, Munich, Germany). The electric field was monitored by the LSProbe 1.2 Field Probe System (LUMILOOP GmbH, Dresden, Germany).

**Figure 1:**
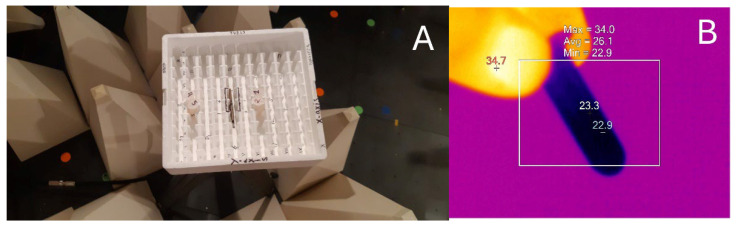
(**A**) The 2.45 GHz EMF exposure setup of the samples in the semi-anechoic chamber. The top of the dipole antenna is visible in the middle of the Styrofoam box where the samples are placed 2 cm (tubes labeled as “1” and “2”) and 4 cm away from the antenna (tubes “4” and “5”). EMF-dissipative cones are also visible. (**B**) Thermogram of a sample obtained by an infrared camera used to monitor temperature change during the treatment period both for irradiated and control samples. False color temperature scale where white corresponds to the highest and black to the lowest temperature, in an arbitrarily defined range. Values for different markers are displayed. Temperatures at the bottom of the tubes where the suspension is located were used for analyses.

For antennas shorter than half of the EMF wavelength (λ), the near-field region extends up to a λ from it. It is further divided into two zones: reactive and radiative. The border between them lies λ/2π away from the antenna. This means that in the described setup, both applied distances were in the near-field, since for a 2.45 GHz EMF wave, λ in air is 12.23 cm, with the antenna length (3 cm) being shorter than λ/2 = 6.12 cm. The spot at 2 cm is just at the transition between the reactive and radiative zones occurring at 1.95 cm, and the 4 cm spot falls well into the radiative zone of the near-field. The exposure setup is presented in Figure 1A.

The specific distances of 2 and 4 cm between the antenna and the yeast samples fall in the distance range between various wearable wireless devices employing the 2.45 GHz EMF band, such as smart phones, smartwatches, smart bands, smart keychains and smart glasses, and the human body. The time intervals of 20 and 60 min resemble the duration of long smartphone calls. Thus the values of both parameters were chosen to reflect conditions observed in the everyday use of smart devices.

For each irradiation setup, the absorbed EMF dose was estimated by calculating the SAR from the microwave heating of the samples during short, 30 s exposure to minimize the effect of heat dissipation. The calculation takes into account the temperature difference (ΔT) between the temperature recorded right after the end of treatment (T_max_) and the initial temperature—room temperature (RT)—and the time duration of exposure in seconds (t = 30 s); the specific heat capacity at constant pressure (c_p_) used in the calculations is that of liquid water—4184 J·kg^−1^·°C^−1^—and is described by the following formula:

SAR = c_p_ · ΔT/t, (1)

where

ΔT = T_max_ − RT. (2)

The sample temperatures were monitored by infrared camera FLIR E5 and the thermograms were analyzed by SmartView Classic 4.4 software (Teledyne FLIR LLC, Arlington, VA, USA) (Figure 1B). Temperatures at the bottom of the tubes where the suspension is located were used for analyses.

Samples that stayed in the semi-anechoic chamber but were wrapped in aluminum foil several times to shield them from the EMF were used as unexposed controls. They were placed at the opposite end of the chamber to exclude EMF reflection to the irradiated samples. In the semi-anechoic chamber, an external EMF is practically missing so the control and treated samples differed only in the presence of (or lack of) irradiation with the generated 2.45 GHz EMF on them. The effective attenuation of the generated EMF by the foil was confirmed by registering a lack of significant temperature rise during the treatment period in the control samples probed by the infrared camera (see Table 1).

Further, for each EMF irradiation variant, two additional conditions were examined to account for the effect of microwave heating: samples held at RT and samples undergoing the temperature difference (ΔT) characteristic of the concrete full-term EMF treatment by conventional heating in a PCH-2 Grant-bio dry block (Grant Instruments Europe B.V.). In fact, for the irradiated samples, the ΔT results from the balance between the energy inflow due to the absorbed EMF and the outflow due to ambient heat transfer to the environment. The ΔT for the conventional heating was applied at equal portions per minute; that is, a constant temperature gradient (ΔT/t) was used. Both sample variants were prepared right after EMF exposure, since T_max_ could be obtained only at its end. Room temperature incubation and conventional heating were performed for the time duration of the particular EMF treatment.

### 2.3. Determination of Cellular Effects of EMF and Heating

The effects of EMF exposure and the corresponding conventional heating on *S. cerevisiae* were examined by probing cell membrane permeability, antioxidant status and genetic material integrity. The permeability of the membrane was determined by the leakage of UV-absorbing substances from the cells during and after treatment. More specifically, the standard measure of absorbance at 260 nm (A_260_) for nucleic acids’ relative concentrations was used to determine their leakage [18]. From each 2 mL sample, 1 mL of yeast suspension was taken, and those samplings were centrifuged at 12,000 RPM for 2 min (Minispin, Eppendorf, Hamburg, Germany) to sediment the cells and separate them from the medium. The supernatants (containing just medium) were collected, and their A_260_ was determined.

The cell antioxidant status was evaluated by the reduced glutathione (GSH) content and Trolox equivalent antioxidant capacity (TEAC) of cell lysates. They were prepared by mechanical disintegration of the cells with 0.666 g quartz sand added to 1 mL of the yeast suspensions at 2700 RPM for 10 min in Digital Disruptor Genie (Scientific Industries Inc., Bohemia, NY, USA). The lysates were centrifuged at 12,000 RPM for 2 min (Minispin) and the supernatant was extracted for analyses. GSH content was determined by A_412_ of TNB chromophore produced by the reaction of GSH with DTNB (D8130, Sigma Chemical Co., St. Louis, MO, USA) [19]. Reduced glutathione (120000050, Thermo Scientific Chemicals, Waltham, MA, USA) was used as standard in the 0–20 μg/mL concentration range. TEAC was established following the ABTS (194430, Sigma-Aldrich Co., St. Louis, MO, USA) radical cation decolorization assay, improved by Re et al., measuring A_734_ [20]. Trolox (218940010, Thermo Scientific Chemicals, Waltham, MA, USA) was used as standard in the 0–17.5 μmol/L concentration range. All photometric analyses were performed with an ONDA UV-21 spectrophotometer (Giorgio Bormac S.r.l., Carpi, Italy).

DNA integrity were determined by single-cell gel electrophoresis in alkaline conditions as per Azevedo et al. and defined as a yeast comet assay [21]. Images of the comets were obtained by an Axioscope 5 microscope, provided with a Colibri 3 imaging system, Axiocam 202 mono camera and ZEN 3.4 (blue edition) program (Carl Zeiss Microscopy GmbH, Jena, Germany). The images were analyzed with CometScore 2.0 software (TriTek Corp., Sumerduck, VA, USA). The following parameters were chosen as measures of DNA damage:

1. Comet tail length (TL, pixels)—comet head diameter subtracted from the overall comet length;
2. Comet tail DNA percentage (TDC, %)—total comet tail intensity divided by the total comet intensity, multiplied by 100;
3. Comet olive tail moment (OTM, arb. u.)—summation of each tail intensity integral value, multiplied by its relative distance from the center of the head (the point at which the head integral is mirrored), and divided by the total comet intensity [22].

All analyses were performed 2 h after treatments finished.

### 2.4. Statistical Analyses

The data presented are means with standard error of mean (SEM) values calculated from 4 independent experiments for the first EMF treatment setup, 2 for the second and 3 for the third. Unless otherwise stated, two-way ANOVA with the experimental variant and experimental repetition factors, followed by the Holm–Sidak test, was used to differentiate statistically significant differences (*p* < 0.05) among variants from those due to variations among individual experiments. The SigmaPlot 11 program (Systat Software, Inc., San Jose, CA, USA) was used. The raw data can be found in Table S1.

## 3. Results

### 3.1. SAR and Microwave Heating

The SAR along with information about the microwave heating of the 2 mL 100 mg FW/mL yeast suspensions treated with a 2.45 GHz EMF for all the exposure conditions are presented in Table 1. The SAR at 4 cm was just 5% lower than that at 2 cm—both around 130 W/kg. While the SAR was almost the same, the microwave heating was significantly different among the applied setups. At the position nearest to the antenna, the temperature rose the most, by 23 °C to T_max_ of 44 °C, which is well above the temperature optimum for the growth of the yeast strain used (30 °C) and might have easily induced heat stress. When the distance increased twice while the duration stayed unchanged (20 min), the temperature difference dropped almost threefold to 8 °C, and the reached T_max_ (32.5 °C) was near the optimum. Increasing the treatment period three times at a 4 cm distance raised the temperature amplitude by 58% to 12.5 °C. It is important to note that the room temperatures were practically the same for all setups.

### 3.2. Cell Membrane Permeability

**Figure 2:**
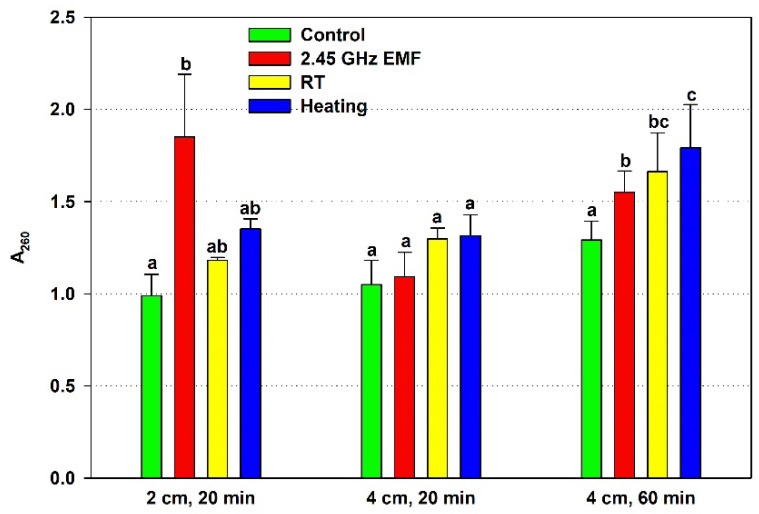
Absorbance at 260 nm (A_260_) determined by the nucleic acids leaked from yeast cells: irradiated with a 2.45 GHz microwave in three exposure conditions, 2 cm away from the antenna for 20 min, 4 cm for 20 min and 4 cm for 60 min (EMF), undergoing corresponding conventional heating (heating), not irradiated (control) or held at room temperature (RT) for the same periods of time. The values presented are mean ± SEM, and distinct letters denote significantly different variants among each experimental setup (*p* < 0.05).

The effect of the 2.45 GHz EMF on yeast cell membrane permeability was assessed by the leaked nucleic acids from the cells, measured as A_260_, and was compared to the effect of conventional heating causing the temperature rise registered for the particular exposure condition (Figure 2). The 20 min irradiation at 2 cm resulted in increased cell leakage almost two-fold (by 86% on average) while the heating (ΔT = 22.8 °C) alone caused an insignificant 16% rise. The 4 cm, 20 min EMF exposure did not change the membrane permeability relative to the not irradiated control, nor did the corresponding conventional heating (ΔT = 7.9 °C) compared to the RT sample. The 60 min irradiation elevated membrane permeability mildly but significantly, by 15% on average. The corresponding conventional heating (ΔT = 12.5 °C) failed to show significant rise, at just 8%.

### 3.3. Cell Antioxidant Status

**Figure 3:**
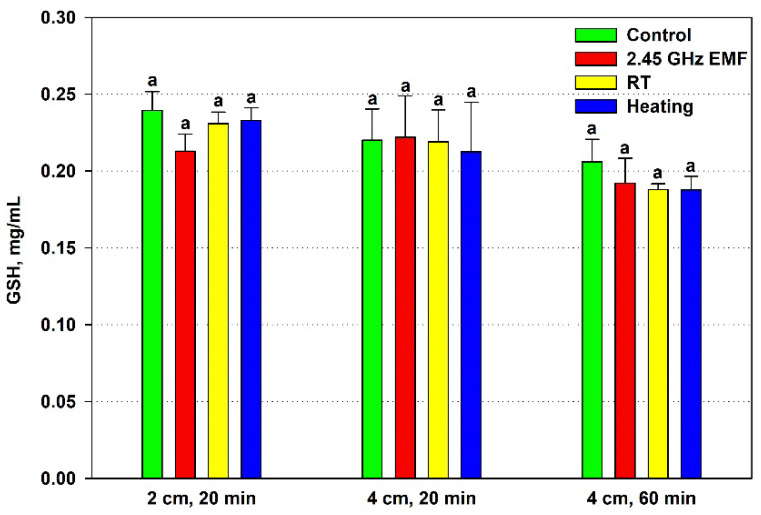
Reduced glutathione (GSH, mg/mL) content in yeast cells: irradiated with a 2.45 GHz microwave in three exposure conditions, 2 cm away from the antenna for 20 min, 4 cm for 20 min and 4 cm for 60 min (EMF), undergoing corresponding conventional heating (heating), not irradiated (control) or held at room temperature (RT) for the same periods of time. The values presented are mean ± SEM, and distinct letters denote significantly different variants among each experimental setup (*p* < 0.05).

The effect of EMF exposure on the yeast cell antioxidant status was assessed by the GSH content and TEAC, comparing it to the effect of the conventional heating. The GSH concentration did not change significantly in any of the applied experimental conditions (Figure 3). Nonetheless, a clear deviation was registered after 20 min irradiation at a 2 cm distance, when GSH declined by 11% on average relative to the control. For comparison, corresponding heating lowered the mean GSH value by just 1% in relation to the RT sample.

**Figure 4:**
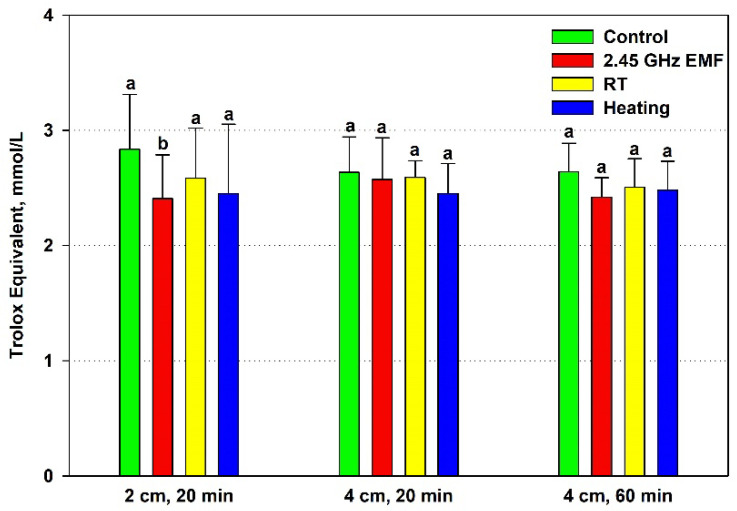
Antioxidant capacity, expressed as Trolox equivalent (mmol/L), of yeast cells: irradiated with a 2.45 GHz microwave in three exposure conditions, 2 cm away from the antenna for 20 min, 4 cm for 20 min and 4 cm for 60 min (EMF), undergoing corresponding conventional heating (heating), not irradiated (control) or held at room temperature (RT) for the same periods of time. The values presented are mean ± SEM, and distinct letters denote significantly different variants among each experimental setup (*p* < 0.05).

The TEAC of the yeast cells decreased significantly during 2 cm, 20 min irradiation (15%) but not under heating by 22.8 °C (Figure 4). The 4 cm distance and temperature rise of 7.9 °C and 12.5 °C did not alter antioxidant activity in a significant way.

There is a clear correlation between the lowered antioxidants and the increased permeability in the 2 cm (132 W/kg) EMF treatment for 20 min, suggesting a common factor determining them. Oxidative stress could have occurred during that exposure. The generated oxidants partially depleted the antioxidants while simultaneously oxidizing lipids, causing membrane fluidization or even disintegration and leading to high leakage. Furthermore, the suggested oxidative stress seems to be unrelated to the temperature rise during irradiation since it does not occur under conventional heating. Interestingly, the permeabilization at 4 cm (126 W/kg) exposure for 60 min seems to have a different mechanism because it is not related to the antioxidant status, yet heating alone has no influence on it.

### 3.4. DNA Integrity

**Figure 5:**
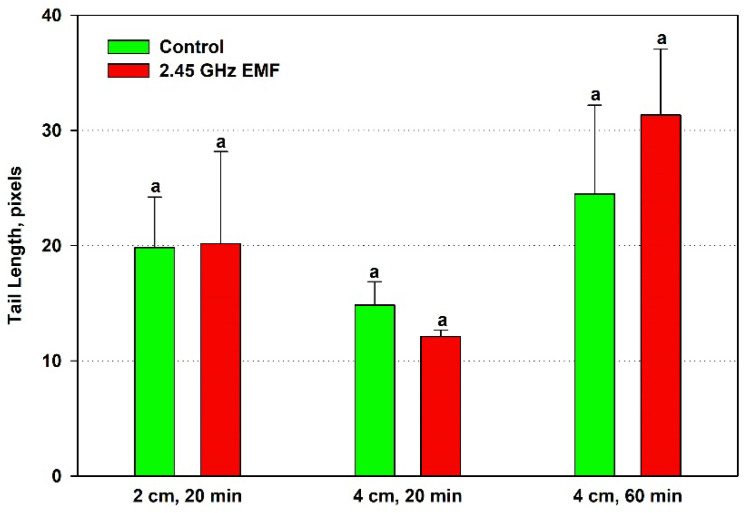
Comet tail length (pixels) determined by single-cell gel electrophoresis of yeast cells irradiated with a 2.45 GHz microwave in three exposure conditions, 2 cm away from the antenna for 20 min, 4 cm for 20 min and 4 cm for 60 min (EMF), or not irradiated (control). The values presented are mean ± SEM, and distinct letters denote significantly different variants among each experimental setup (*p* < 0.05).

The effect of EMF exposures on the yeast DNA was assessed by three comet assay parameters—comet tail length, comet tail DNA percentage and comet olive tail moment. Tail length increased by just 2% from control levels on average after the 20 min treatment at 2 cm, while at 4 cm, it decreased by 19% (Figure 5). During 60 min exposure, the biggest difference between the mean values for control and treated samples was observed—28% in favor of the EMF. However, all those changes were not statistically significant.

**Figure 6:**
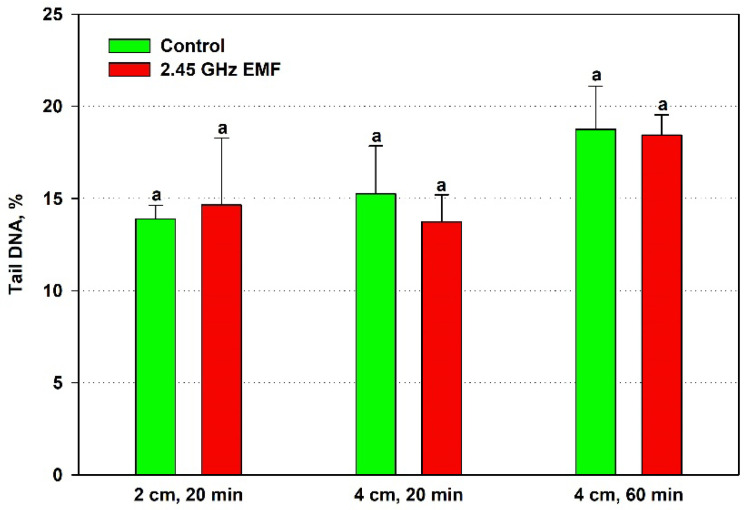
Comet tail DNA percentage determined by single-cell gel electrophoresis of yeast cells irradiated with a 2.45 GHz microwave at three exposure conditions, 2 cm away from the antenna for 20 min, 4 cm for 20 min and 4 cm for 60 min (EMF), or not irradiated (control). The values presented are mean ± SEM, and distinct letters denote significantly different variants among each experimental setup (*p* < 0.05).

The alterations in comet tail DNA percentage were even less pronounced than those in tail length, so it is not surprising that no statistically significant differences were revealed for it either (Figure 6). The biggest discrepancy was found at 2 cm, 20 min irradiation—the average levels of the EMF-exposed samples were 10% lower than controls.

**Figure 7:**
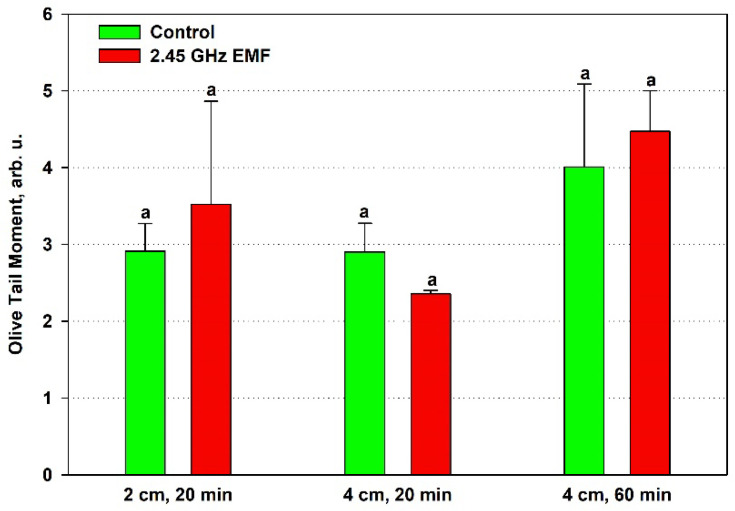
Comet olive tail moment (arb. u.) determined by single-cell gel electrophoresis of yeast cells irradiated with a 2.45 GHz microwave at three exposure conditions, 2 cm away from the antenna for 20 min, 4 cm for 20 min and 4 cm for 60 min (EMF), or not irradiated (control). The values presented are mean ± SEM, and distinct letters denote significantly different variants among each experimental setup (*p* < 0.05).

The olive tail moment displayed an average increase of 21% at 2 cm and 12% at 4 cm for the 60 min treatment but a decrease of 19% at the shorter period compared to the respective control (Figure 7). Despite the fact that the OTM is expected to be the most sensitive of the three comet assay parameters since it summarizes the information of the other two, the data variance was not improved and random sampling variability could not be excluded as a reason for the observed differences.

Despite the lack of statistical significance for the differences obtained in the studied comet assay parameters, a trend towards an increased amount of DNA damage was observed in the samples placed 2 cm away from the antenna that absorbed 132 W/kg during the shorter 20 min period and those at a 4 cm distance (126 W/kg) for 60 min, as visible from Figure 7, where the mean OTM values in the EMF-treated samples are higher than in the controls for both conditions. In the first case, this was expected, as that was the only treatment condition in which strong oxidative stress was implied by the high membrane permeability and the antioxidant activity drop. It is interesting that at the lower SAR irradiation, the shorter 20 min period indicated increased DNA integrity, as if repair mechanisms were stimulated. However, that stimulation should have been transient since the longer period seemed to allow the accumulation of DNA breaks that the cells’ repair systems were unable to fix.

## 4. Discussion

The most profound EMF effect revealed in our study is the strong disruption of cell membrane integrity at 132 W/kg, as demonstrated by the large nucleic acid release from the yeasts. It is known that a significant release of intracellular substances can only be observed after reversible or irreversible permeabilization and that the type and amount of these substances depend on the defects in the cell membrane [23,24]. Our finding is in concordance with previous reports of increased membrane permeability under EMF irradiation [12]. The corresponding conventional heating failed to demonstrate significant leakage, hinting at a non-thermal mechanism of EMF action on membrane integrity. Such a mechanism was implied by Nguyen et al., describing a possible EMF-induced mechanical disturbance altering the membrane tension permeability and thus enhancing the degree of membrane trafficking through the lipid bilayer [25]. Researchers have reported non-thermal effects of EMFs on other biological processes as well: glucose uptake by yeast cells [26], cell proliferation [4] and cell growth [27].

Alternatively, the vast discrepancy between the observed results for heating and EMFs might not reveal the presence of non-thermal effects under EMFs, but could be explained by the lack of effects under conventional heating. The lack of significant permeabilization under heating to 44 °C might seem puzzling by itself since at that temperature protein denaturation and increased fluidity of cell membranes are highly plausible [28,29]. However, it should be noted that the temperature gradient provided by the heater was constant, leading to a linear temperature increase, meaning that the 44 °C is reached just at the end of the treatment and not sustained for more than a minute. On the other hand, it is highly possible that the temperature rise caused by EMF radiation was not linear throughout the exposure period—the maximal temperature could be reached until a thermal equilibrium with the environment is reached before the treatment end, followed by a prolonged period during which that temperature is maintained. If that was the case, the effective temperature during exposure is definitely higher than during conventional heating, which could explain the strong EMF effect in terms of a thermal mechanism. It is well known that temperature increases membrane fluidity leading to defects through which substances are released [30,31,32]. Another highly possible supposition is that yeast autolysis developed—a process characterized by a loss of cell membrane permeability at first, alteration of cell wall porosity, hydrolysis of cellular macromolecules by endogenous enzymes and subsequent leakage of the breakdown products into the extracellular environment, known to be induced by elevated temperatures (40–60 °C) [33]. Indeed, for a particular temperature difference, microwave heating of a water-based solution is faster than conventional heating because it is based on the direct transfer of EMF energy at the molecular and the nano-cluster scale of the water structure without depending on heat transfer [34]. This defines microwave heating as internal, as opposed to conventional external heating. Moreover, microwave heating has characteristic properties such as selectivity, hot spot formation, local effects and nonuniformity. All those make imitating it by conventional methods, i.e., external heat transfer, practically impossible. However, in the irradiation setup applied in the study, relatively uniform EMF heating of the samples could be assumed since the tube diameter (1 cm) was smaller than the penetration depth of the 2.45 GHz microwaves, 1.8 cm for water at 25 °C, and increasing with temperature. It was previously proposed that internal ‘micro’-thermal effects specific to microwave radiation may underpin specific biological effects on membranes, proteins, enzyme activity and cell death that cannot be explained by virtue of temperature increases alone [35].

The 60 min exposure at 126 W/kg distinctly increased the release of UV-absorbing components from the cells but was much less pronounced than the 132 W/kg treatment, while the corresponding conventional heating did not cause significant leakage yet. The maximum temperature reached was 36.6 °C, which is within the physiological range for yeast growth, not implying autolysis but a specific EMF effect on the membrane. A similar finding was reported by Ahortor et al. when the electrical component of an electromagnetic field at a frequency of 2.45 GHz increased the membrane permeability of yeast cells to certain molecules, such as propidium iodide, but thermal treatment at 37 °C did not [13]. However, leakage under 60 min irradiation could also be explained by a higher effective temperature under microwave than conventional heating due to a faster temperature rise to maximal temperature. Moreover, even if the temperature rise could not induce membrane defects big enough to compromise integrity, it can definitely boost the quantity of the leaked substances since they are released by diffusion, which depends exponentially on temperature [36].

When comparing our study with similar studies on yeast [12,13], the large differences in irradiation settings (the parameters of the applied EMF) and exposed biological samples do not allow a reasonable comparison of results. In those studies, various irradiation parameters were examined—cell survival, ROS, membrane permeability for different sized particles, DNA release and morphological changes in the cell surface—but they lack data on the antioxidant status of the cells after treatment, as well as on the impact of the EMF on the genetic material. However, the decreased amount of antioxidants after irradiation that we found correlates with the increased levels of ROS in the study by Riffo et al. [12]. So, in agreement with the other two studies, we established (1) increased membrane permeability and (2) elevated oxidative stress as a result of the 2.45 GHz EMF action, thus confirming both effects as the two of the main mechanisms of cell damage after microwave EMF irradiation.

Stress at the cellular level is often manifested as an imbalance between oxidants, mainly ROS, and antioxidants in favor of the former—oxidative stress [37]. Therefore we examined the quantity of intracellular antioxidants—reduced glutathione and total antioxidant capacity of yeast cells. The only proven effect was observed for the 132 W/kg EMF, where TEAC dropped, while a trend towards a decreased amount of GSH in the cells was also noticed. Both observations could be explained by oxidative stress developing under irradiation. On the other hand, the 44 °C temperature achieved during conventional heating did not affect neither TEAC nor GSH despite the fact that increased levels of ROS have been observed in yeast cells subjected to heat stress [38]. Elevated temperatures can impair mitochondrial function, leading to increased electron leakage from the respiratory chain and subsequent ROS production [39]. Therefore, the observed antioxidant status changes in the yeast suspensions induced specifically by the EMF might be due to non-thermal effects. Indeed, oxidative stress is one of the proposed non-thermal mechanisms of action of EMFs on organisms [40]. However, higher heating during irradiation cannot yet be ruled out as a plausible cause.

Our study failed to demonstrate a clear genotoxic effect of the applied EMF. Just weak tendencies towards increased DNA damage at 132 W/kg for 20 min and at 126 W/kg for 60 min were indicated. Some previous studies have found potential genotoxicity of 2.45 GHz EMFs under certain treatment conditions in specific cell types [41], while others have not found a damaging effect [42,43]. The generated reactive oxygen species during irradiation might underlie such DNA damage [44]. Different cell types exhibit different DNA sensitivity to EMFs. Neuronal-like SH-SY5Y cells show increased levels of ROS and mitochondrial dysfunction under exposure, indicating increased sensitivity compared to peripheral blood mononuclear cells [11]. In a study of 905 MHz radiation on different strains of *S. cerevisiae*, it was found that strains deficient in DNA repair mechanisms showed significantly reduced colony growth compared to wild-type strains, suggesting DNA breaks [45]. There is evidence that carrier frequency EMFs with a typical modulation structure of the GSM signal affect DNA in human trophoblast cells, causing a transient increase in the level of DNA fragmentation that disappears within 30 to 120 min [46]. So, since our samples were incubated for 2 h after irradiation at room temperature prior to analysis, transient DNA damage could have been fixed by the repair mechanisms, thus explaining the inconclusive results obtained. It has been found that in yeast, about 80% of single-strand DNA breaks are repaired very quickly, within 3 min, and the remaining 20% more slowly, within 20 min, which leads to almost complete repair of the damage, but this happens in a nutrient medium; in water, this process is delayed [47]. Repair of double-stranded breaks takes longer: the first repair products appear 1–2 h after the break, but it can take even longer [48]. However, there are no studies that have reported DNA damage in yeast after exposure to 2.45 GHz, although similar studies have been conducted in other organisms or at other frequencies. Scientists have found single-strand and double-strand DNA breaks in rat brain cells, 4 h after 2 h irradiation with a 2.45 GHz EMF (SAR 1.2 W/kg for whole body), as measured by a comet assay. The authors suggest that the damage may be a result of the direct effects of the EMF on DNA molecules and/or disruption of DNA repair mechanisms in brain cells [10].

There are some limiting factors in our study, explaining the lack of definitive results. The number of experimental repetitions is insufficient to obtain statistically significant differences in some of the studied parameters (e.g., DNA damage) or to completely reject possible effects. To clearly distinguish thermal from non-thermal effects of EMFs, a more precise determination of the effective temperature during treatment and a corresponding reproduction of such heating are necessary. A wider range of studied biological parameters is also needed to gain a comprehensive picture of the yeast cell response to the applied EMF, which would be the subject of future research.

Future studies in this direction could focus on longer-term or repeated irradiations with weaker EMFs, while not allowing the temperature of the irradiated sample to increase in order to avoid the thermal effects of microwaves. Thus, the treatment conditions would be closer to those to which people are exposed daily. In this way, any effects observed as a result of the treatment would be exclusively non-thermal. This would be of great importance for a better understanding of the impact of the EMF on the cell membrane to study its fluidity after or even during irradiation, as well as lipid peroxidation, in order to establish the specific cause of the increased release of substances. Subsequent studies could be carried out using yeast strains with deficient antioxidant systems, as well as assessing gene expression responses related to oxidative stress and the activity of antioxidant enzymes. In order to detect DNA damage and repair processes, it would be good to conduct a comet assay at a shorter period after irradiation, for example, 30 min, and in a time series, e.g., at 60 min intervals for several hours.

## 5. Conclusions

The EMF exposure conditions we applied did not cause significant damage to the yeast cells. When irradiating a sample at a distance of 2 cm from the antenna for 20 min, increased permeability of the cell membrane was observed, determined by the elevated release of UV-absorbing substances from the cells. Under the same conditions, a decrease in antioxidant capacity was reported, suggesting the development of oxidative stress under treatment. Samples heated in a conventional manner to the temperature reached during irradiation did not show similar changes, hinting at plausible non-thermal effects revealed. With the longer irradiation for 60 min at a greater distance (4 cm), a slight increase in membrane permeability was found, but without changes in antioxidant status. No statistically significant DNA damage was found in any of the irradiated samples. Future studies are needed to unequivocally elucidate the mechanism underlying the 2.45 GHz EMF effects on yeast and eukaryotic cells as a whole.

## Figures and Tables

**Table 1:** SAR and temperatures for yeast suspensions treated with 2.45 GHz EMF at different exposure conditions and the respective controls.

Exposure Setup	Sample Type	Distance to Antenna, cm	Duration, min	SAR, W/kg	RT, °C *	T_max_, °C *	ΔT, °C *
1	EMF	2	20	132	21.4 ± 0.8 ^a^	44.2 ± 0.7 ^a^	22.8 ± 1 ^a^
1	Control	N.A.	20	N.A.	21.5 ± 0.7 ^a^	24.2 ± 0.2 ^d^	2.7 ± 0.7 ^d^
2	EMF	4	20	126	24.6 ± 1.5 ^a^	32.5 ± 2.3 ^c^	7.9 ± 0.8 ^c^
2	Control	N.A.	20	N.A.	24.5 ± 1.6 ^a^	25.8 ± 0.9 ^d^	1.4 ± 0.8 ^d^
3	EMF	4	60	126	24.2 ± 0.7 ^a^	36.6 ± 1.2 ^b^	12.5 ± 0.4 ^b^
3	Control	N.A.	60	N.A.	24.3 ± 0.9 ^a^	27.2 ± 0.3 ^d^	2.9 ± 0.7 ^d^

* The values presented are mean ± SEM from the experimental repetitions for each setup, and distinct letters denote significant differences among all experimental conditions (*p* < 0.05). One-way ANOVA followed by the Holm–Sidak test were performed. N.A.—not applicable.

## Data Availability

The original contributions presented in this study are included in the article/Supplementary Materials. Further inquiries can be directed to the corresponding author.

## Supplementary Materials

The following supporting information can be downloaded at: https://www.mdpi.com/article/10.3390/antiox14070820/s1, Table S1: Raw data.

## Abbreviations

The following abbreviations are used in this manuscript:

A_260_	Absorbance at 260 nm
A_412_	Absorbance at 412 nm
A_734_	Absorbance at 734 nm
ABTS	2,2′-azino-bis(3-ethylbenzothiazoline-6-sulfonic acid)
DTNB	5,5-dithio-bis-(2-nitrobenzoic acid) (Ellman’s Reagent)
d. w.	Distilled Water
EMF	Electromagnetic Field/s
FW	Fresh Weight
GSH	Glutathione (reduced form)
N.A.	Not Applicable
ROS	Reactive Oxygen Species
RPM	Revolutions Per Minute
RT	Room Temperature
SAR	Specific Absorption Rate
TEAC	Trolox Equivalent Antioxidant Capacity
T_max_	Temperature (maximal) recorded right after the end of treatment
YPD	Yeast extract, Peptone, Dextrose
ΔT	Temperature difference between T_max_ and RT
λ	Wavelength
